# An Unsupervised Multichannel Artifact Detection Method for Sleep EEG Based on Riemannian Geometry

**DOI:** 10.3390/s19030602

**Published:** 2019-01-31

**Authors:** Elizaveta Saifutdinova, Marco Congedo, Daniela Dudysova, Lenka Lhotska, Jana Koprivova, Vaclav Gerla

**Affiliations:** 1 Czech Technical University in Prague, Jugoslávských partyzánů 1580/3, 160 00 Prague, Czech Republic; lenka.lhotska@cvut.cz (L.L.); vaclav.gerla@cvut.cz (V.G.); 2 National Institute of Mental Health, Topolová 748, 250 67 Klecany, Czech Republic; daniela.dudysova@nudz.cz (D.D.); jana.koprivova@nudz.cz (J.K.); 3 GIPSA-lab, University Grenoble Alpes, CNRS, Grenoble-INP, 38000 Grenoble, France; marco.congedo@gipsa-lab.fr; 4 Charles University, Third Faculty of Medicine, Ruská 2411/87, 100 00 Prague, Czech Republic

**Keywords:** sleep EEG, artifact detection, Riemannian geometry

## Abstract

In biomedical signal processing, we often face the problem of artifacts that distort the original signals. This concerns also sleep recordings, such as EEG. Artifacts may severely affect or even make impossible visual inspection, as well as automatic processing. Many proposed methods concentrate on certain artifact types. Therefore, artifact-free data are often obtained after sequential application of different methods. Moreover, single-channel approaches must be applied to all channels alternately. The aim of this study is to develop a multichannel artifact detection method for multichannel sleep EEG capable of rejecting different artifact types at once. The inspiration for the study is gained from recent advances in the field of Riemannian geometry. The method we propose is tested on real datasets. The performance of the proposed method is measured by comparing detection results with the expert labeling as a reference and evaluated against a simpler method based on Riemannian geometry that has previously been proposed, as well as against the state-of-the-art method FASTER. The obtained results prove the effectiveness of the proposed method.

## 1. Introduction

Human electroencephalography (EEG) refers to recordings of brain activity obtained using electrodes placed on the surface of the head. EEG investigation is one of the main research methods in sleep medicine. It aims at recording electrical activity caused by internal processes during pre-sleep wakefulness and sleep. The number of electrodes and their locations are set according to the areas of interest. Modern sleep research often utilizes at least 19 channels distributed evenly over the head. Some recent studies have applied up to 256-channel high-density EEG [1,2]. Manual data inspection is a demanding and tedious task, especially in long-term recordings such as a whole-night EEG. This makes automatic processing methods highly needed in sleep medicine research.

**Figure 1:**
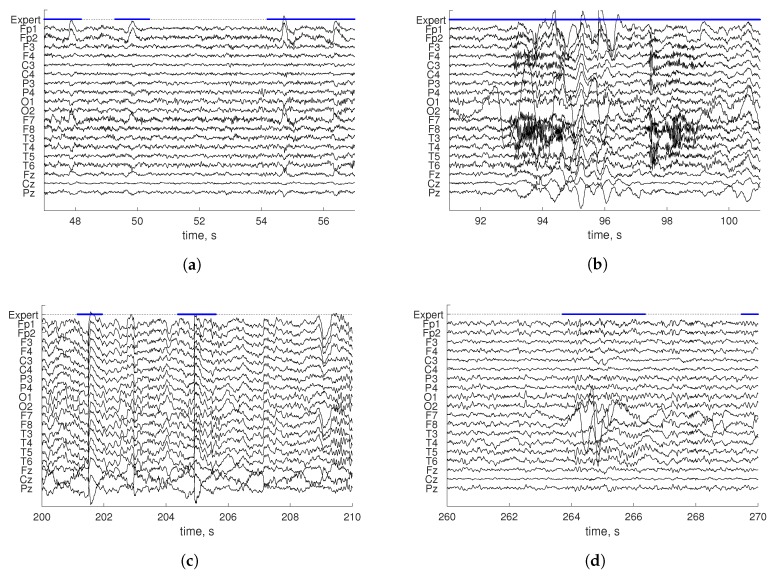
Expert labeling (Expert) of blinking artifacts (**a**), movement artifact during wakefulness (**b**), and electrode artifacts in non-REM 3 (**c**) and REM (**d**). Solid blue lines are the expert’s artifact marks.

Events not related to brain activity may noticeably affect automatic analysis or make it even impossible. Such events are called artifacts. Typical causes of artifacts are technical issues (detached electrodes, sweating, electrical noise) and physiological events (muscle contractions, movements, eye rolling) of the body. For instance, artifacts caused by blinking are characterized by slow-frequency/high-amplitude waves that can be seen mainly in frontal electrodes. The typical waveform corresponding to such artifacts is shown in Figure 1a. Blinking occurs only during the awake state. Artifacts associated with movements may arise during both being awake and asleep, the latter divided into rapid eye movement (REM), non-REM 1, 2, and 3 sleep stages [3]. Such artifacts are characterized by an unusually high amplitude and unusual frequency in several electrodes, as is shown in Figure 1b. Electrode artifacts may arise during the whole recording, and they may have different characteristics. Voltage jumps could be seen in all channels and are illustrated in Figure 1c. A loss of contact of electrodes may produce noise in a channel or high amplitude anomalies, as shown in Figure 1d.

Manual artifact detection is a time-consuming task requiring specific skills. Manual detection is more difficult in high density EEG recordings (128 or 256 channels), and it might lead to a higher number of undetected artifacts on one side and to false detection on the other side. There is a wide choice of automated artifact detection methods available in the literature [4]. They include approaches based on signal decomposition such as Fourier transform [5], wavelet transform [6,7], or empirical mode decomposition [8,9]. A popular method for eye blinking artifact detection is regression [10,11]. Blind source separation (BSS) methodologies like independent component analysis (ICA) [4,5,12] and independent vector analysis [13] are often used for ocular, muscular, and cardiac artifact rejection. Once the decomposition has been achieved, components corresponding to artifact activity may be rejected manually or using automatic methods [14]. A combination of BSS and other signal decomposition techniques may bring benefits for muscular artifact removal [15,16]. Many recent studies have proposed methods based on classification techniques such as random forest classifiers [7], artificial neural networks [17], or support vector machine classifiers [18,19], where training examples determine the types of artifacts to be detected. Another direction of modern artifact detection is the optimization of existing approaches [20,21], adjusting them to the increasing volume of recorded data. Research in the field of sleep data processing mainly focuses on the detection and elimination of the most expected artifacts during sleep [22] through single-channel approaches [23]. These methods concentrate only on certain artifact types. Therefore, artifact-free data are obtained after sequential application of different methods, and single-channel approaches must be applied to all channels alternately.

The aim of the study is to develop an artifact detection method in multichannel sleep EEG capable of rejecting different artifact types at once. The inspiration for the study is gained from the idea of the Riemannian potato [24], an automatic and adaptive artifact detection method proposed for online brain-computer interface (BCI) experiments. It considers spatial patterns of awake EEG data and exploits the fact that clean data tend to form a cluster in the manifold of symmetric positive matrices (the “potato”), the space on which the EEG data is mapped, while artifacts tend to be distributed outside the cluster. In our study, we assume that the number of clusters for clean data may be superior to one, and we introduce an unsupervised and adaptive method for constructing the clusters. It allows us to achieve better performance for complex data such as sleep EEG, where we cannot assume a unique cluster because the spatial patterns vary widely across sleep stages and because sleep data within the same stage may be captured more accurately by several clusters. Different sleep stages have their own characteristics. In the present study, we investigate the proposed method and test it on real sleep EEG data. Performance is evaluated by comparing the obtained detection to a provided expert scoring. Results are examined in contrast to the outcome of the (one-cluster) Riemannian potato artifact detector [24] and the fully-automated statistical thresholding for EEG artifact rejection method (FASTER) [25], which performs artifact detection by analyzing statistical properties in separate channels.

## 2. Data

### 2.1. Dreams Dataset

The open-source Dreams dataset [26] contains 15-min recordings of sleep data, 20 recordings in total. Channels originally referenced to A1 were re-referenced to Cz. All available EEG channels O1-Cz, O2-Cz, Fp1-Cz, and Fp2-Cz were used in the study. The sampling frequencies were 50 Hz, 100 Hz, and 200 Hz. We excluded from analysis the recordings sampled at 50 Hz due to the fact that with such a sampling rate, the frequency band-pass is too restricted for sleep data analysis [3]. Furthermore, we excluded recording Numbers 3, 6, 10, and 16 because the proportion of artifacts exceeded 40%. Sleep stage scoring and artifact labeling have been performed by a trained clinician and provided, as well. Each recording contains a mix of sleep stages. The distribution of the sleep stages in the dataset is: wakefulness 33%, REM 25%, non-REM 1 18%, non-REM 2 23%, and other 1%. Information on the artifact rate is shown in Table 1.

### 2.2. InSleep Datasets

The InSleep datasets were obtained from the National Institute of Mental Health, Czech Republic. The study was conducted in accordance with the Declaration of Helsinki, and the protocol was approved by the Ethics Committee of the National Institute of Mental Health (Project Number 6/15). The whole-night EEG as a part of the polysomnography (PSG) was recorded from subjects suffering from insomnia. Each recording was obtained using 19 channels placed according to the 10-20 system: Pz, Cz, Fz, T6, T5, T4, T3, F8, F7, O2, O1, P4, P3, C4, C3, F4, F3, Fp2, Fp1. Channels were referenced to an average of Cz, Fz, and Pz. The sampling frequency was 250 Hz. Sleep stages were scored by a trained clinician. Each recording contained 10–20 min of data corresponding to a single sleep stage (REM, non-REM 2, or non-REM 3) or awake activity. The non-REM 1 sleep stage was not presented in the dataset due to its short duration in sleep recordings. Artifacts were labeled by an expert. Visual inspection of EEG was performed separately for each recording in the EEG Lab software using function “reject continuous data by eye” [27]. The expert was instructed to label all visible artifacts. The total number of obtained recordings was 44. Details of the artifact rate are provided in Table 1.

## 3. Materials and Methods

### 3.1. Theoretical Background

This section describes the theoretical aspects of working with multichannel data. We denote by $x_{t}\in R^{n}$ the signal vector at *n* channels and at time point *t*. $X=\left[x_{s},\ldots ,x_{s+m-1}\right]\in R^{n\times m}$ denotes an epoch starting at sample *s* and lasting *m* samples. For *X*, we estimate the spatial covariance matrix using the usual sample covariance matrix (SCM) estimator:

(1) $$C=\frac{1}{m-1}XX^{T}\in R^{n\times n}.$$

The matrix *C* is symmetric positive-definite (SPD). Often, SPD matrices are considered in a Euclidean space with the associated Frobenius norm ${∥C∥}_{F}$. However, the native space of SPD matrices is not Euclidean, but a Riemannian space [28]. Riemannian geometry starts by defining an inner product (metric) at each tangent space to the manifold, which varies smoothly from point to point. Applying the affine-invariant (Fisher) metric [28], the distance δ between two points $C_{i}$ and $C_{j}$ on the manifold is given by:

(2) $$\delta \left(C_{i},C_{j}\right)={\left[\sum_{i=1}^{n}log^{2}\lambda_{i}\right]}^{1/2},$$

where the $\lambda_{i}$ are the eigenvalues of $C_{i}^{-1}C_{j}$.

A centroid (called the geometric mean) of an SPD matrix set can be defined using the above distance [29]. The geometric mean *M* of a covariance matrix set $C_{i}$ is calculated using an iterative algorithm. First, *M* is initialized by the arithmetic mean of $C_{i}$. Then, the following iteration is repeated:

(3) $$M=M^{1/2}\exp\left[1/k\sum_{k}\ln\left(M^{-1/2}C_{k}M^{-1/2}\right)\right]M^{1/2}$$

until convergence, which is obtained when:

(4) $$∥\sum_{k}\ln{(M^{-1/2}C_{k}M^{-1/2})∥}_{F}<\varepsilon .$$

The distance of a set of covariance matrices to their geometric mean does not have a symmetric distribution. To make the distribution symmetric, we may use the standardized distances to the geometric mean [30]; define the geometric mean μ of the distances $\delta_{k}$ as:

(5) $$\mu =\exp(\frac{1}{k}\sum_{k}\ln\delta_{k})$$

and the geometric standard deviation σ of the distances as:

(6) $$\sigma =\exp(\sqrt{\frac{1}{k}\sum_{k}{\left(\ln\frac{\delta_{k}}{\mu }\right)}^{2}}).$$

Then, the standardized distance measures are:

(7) $$\delta_{k}^{\prime }=\frac{\ln\frac{\delta_{k}}{\mu }}{\ln\sigma }.$$

### 3.2. The Riemannian Potatoes’ Artifact Detection

**Figure 2:**
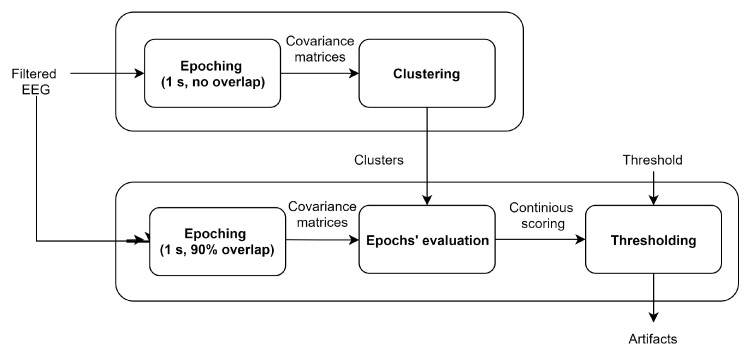
The method overview.

A Riemannian potatoes artifact detection method (RPs) is proposed in this study. It relies on the assumption that on the SPD manifold, contaminated epochs are mapped far away from clusters of clean data. In contrast to the simple potato [24], here we take into consideration the fact that several clusters may be needed to describe the distribution of clean sleep data. In the first step, RPs restore these clusters from a recorded EEG. In the second step, we obtain continuous scoring by analyzing the data in a sliding window and comparing them to the obtained clusters. Finally, artifacts are detected by applying a threshold. The method overview is depicted in Figure 2. As in [24], we filtered the data before further processing. However, we used a low-pass filter under 30 Hz to save frequencies significant for sleep stages [3].

The cluster construction process starts with splitting the filtered data into non-overlapping 1-s epochs. Such an epoch length is a standard for Riemannian geometry analysis [24]. Covariance matrices are extracted from every epoch. Then, distances between all pairs of covariance matrices are calculated by Equation (2). The average distance $d_{i}$ is the mean value of distances from the *i*th epoch to all the other epochs. Epochs with a *d* value greater than a threshold are removed from further analysis. We consider the mean value of *d* as the threshold. Then, we cluster the remaining matrices using the k-means algorithm with the distance measure defined by Equation (2). To determine the number of clusters, we use the following procedure: the number of clusters increases iteratively from one to a maximum of 10; centroids are obtained as the geometric means; the process is finished once the constructed clusters satisfy an overall normality condition, that is when the distribution of standardized distances to the centroids is normal in all clusters. Normality is tested using D’Agostino’s K${}^{2}$ test. The distribution of distances to the centroid within a cluster is examined separately. The overall normality *p*-value is obtained as a combination of the obtained *p*-values using Stouffer’s Z-score method. The cluster construction process is stopped when the combined *p*-value is superior to 0.05. If the cluster construction finishes without achieving overall normality, then we choose the number of clusters achieving the greatest *p*-value. In the case of tied *p*-values, we retain the one corresponding to the smaller number of clusters. The rationale for this procedure is that when the distances of the points in the cluster from the center of mass have a Gaussian distribution, we assume that the cluster is a good representative of a region in the manifold covered by the data and that further splitting is therefore unnecessary.

In the second step, RPs analyze the *n*-channel data in a sliding window of a size of 1 s with a step equal to 0.1-times the sampling frequency. Assume there are *k* clusters obtained in the previous step and denote $O_{j}\in R^{n\times n},j\in \left[1,k\right]$ their centroids. For each *i*th window, we obtain covariance matrix $C_{i}\in R^{n\times n}$. All *k* distances $\sigma_{ij}$ between $C_{i}$ and every $O_{j}$ centroid are calculated. Then, the closest cluster $j^{*}$ to $C_{i}$ is determined by the minimum distance among the $\sigma_{ij}$ values. The geometric mean and standard deviation within the cluster were determined in the cluster construction step. Therefore, we can obtain $\sigma_{i}^{*}$ as a standardized distance $\sigma_{ij^{*}}^{\prime }$ to the centroid $O_{j^{*}}$ by Equation (7). Due to the cluster construction, standardized distances within a cluster are distributed normally. We can consider the obtained $\sigma_{i}^{*}$ as a z-score and translate it into a probability value $p_{i}$ using the Gaussian cumulative distribution function. The value $p_{i}$ indicates a probability of epoch *i* with covariance matrix $C_{i}$ to belong to a given cluster. Hence, the value of $p_{i}^{\prime }$ equals $1-p_{i}$ and indicates a probability of epoch *i* with covariance matrix $C_{i}$ to be an outlier.

We consider that the values $\sigma_{i}^{*}$ correspond to the center of the *i*th epoch and that in an epoch, the scoring starts and ends with 0. Therefore, we interpolate the obtained values within the epoch. The obtained score is then smoothed with a moving average filter to make the output more robust. The local minima of these scores define segments. Finally, a segment whose score is above a threshold for at least 0.4 s is considered as an artifact. This value was established as a minimum artifact duration by the experts.

## 4. Experimental Results

### 4.1. Results Evaluation

Artifact detection performance was evaluated by comparing the detection obtained by the automatic artifact detection methods to a scoring provided by human experts. The comparison was performed on a sample-by-sample basis. We denote the number of true positives, false positives, true negatives, and false negatives as TP, FP, TN, and FN respectively.

**Figure 3:**
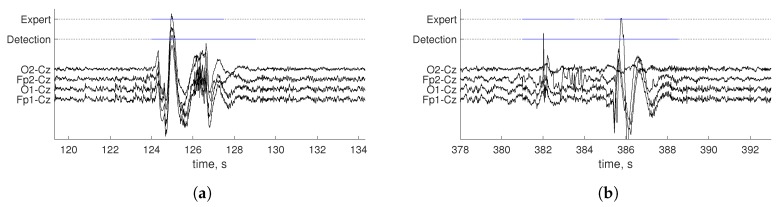
Examples of comparison of human scoring and artifact detection (**a**,**b**).

**Figure 4:**
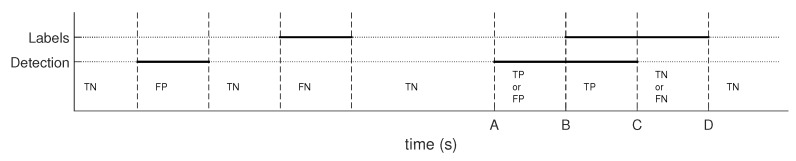
Detection evaluation. Horizontal bold lines represent detected segments. The horizontal dotted line stands for segments labeled as artifact-free data. Vertical dashed lines are the ends of all labeled segments.

Precise artifact borders are hard to determine. Human and automatic scoring may differ and yet be both considered correct, as is shown in Figure 3. To handle this, we added a fuzzy area on each artifact border labeled by an expert. Thus, residual segments with no matching labeling as Segments A-B and C-D in Figure 4 were treated as TP and TN, respectively, if they lasted for at least 10% of the artifact duration and no more than 1.5 s. Otherwise, they were counted as FP or FN, respectively. The parameters used for results evaluation were estimated based on experience and consultancy with neurological experts who described their visual inspection process. 

Following [22], we computed Cohen’s kappa *K* and the percentage of agreement *S*. *K* is considered a more robust measure than *S* because it takes into account agreement occurrence by chance. Values of *K* greater than 0.6 are considered as good and values higher than 0.8 indicate an excellent agreement [31]. We have:

(8) $$K=\frac{P_{o}-P_{r}}{1-P_{r}},$$

where:

(9) $$P_{o}=\frac{\mathrm{TP}+\mathrm{TN}}{\mathrm{TP}+\mathrm{TN}+\mathrm{FP}+\mathrm{FN}},$$

(10) $$P_{r}=\frac{\left(\mathrm{TP}+\mathrm{FN})*(\mathrm{TP}+\mathrm{FP})+(\mathrm{FP}+\mathrm{TN})*(\mathrm{TN}+\mathrm{FP}\right)}{{\left(\mathrm{TP}+\mathrm{TN}+\mathrm{FP}+\mathrm{FN}\right)}^{2}}.$$

Sensitivity Se and false discovery rate (FDR) were also compared in this study.

(11) $$Se=\frac{\mathrm{TP}}{\mathrm{TP}+\mathrm{FN}},$$

(12) $$FDR=\frac{\mathrm{FP}}{\mathrm{TP}+\mathrm{FP}}.$$

The non-parametric Wilcoxon signed rank test was used for comparing metrics. This test does not require normality of the samples and is also suitable for small sample sizes.

### 4.2. Results

**Figure 5:**
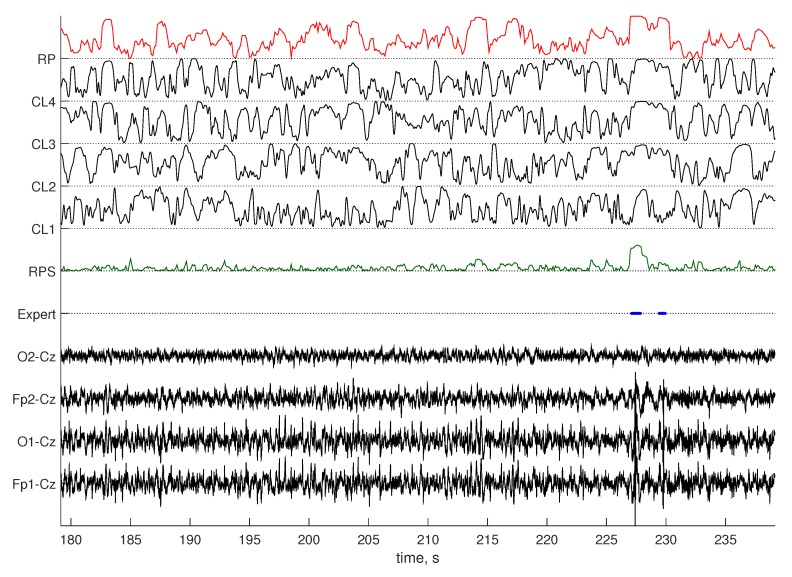
Scoring obtained by the benchmark method (RP) and RPs (RPS) on data from the Dreams dataset. Normalized distances to four clusters obtained for this recording with RPs (CL1–4) are shown, as well. Artifacts (Expert) are denoted with the solid blue line.

**Figure 6:**
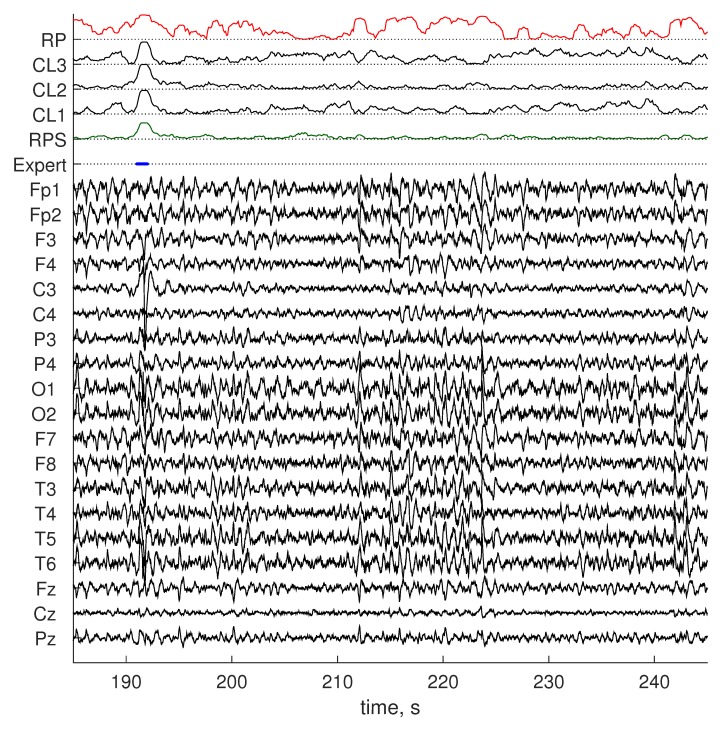
Scoring obtained by the benchmark method (RP) and RPs (RPS) on data from the InSleep dataset. Normalized distances to three clusters obtained for this recording with RPs (CL1–3) are shown, as well. Artifacts (Expert) are denoted with a solid blue line.

**Figure 7:**
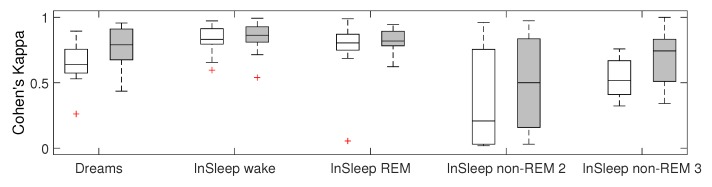
Distribution of Cohen’s kappa for the Dreams and InSleep datasets. White boxes represent the benchmark; grey boxes represent the proposed method.

Our RPs artifact detection method was implemented in Python and applied to the Dreams and InSleep datasets. The Riemannian potato [24] method was chosen as the benchmark. We used the original implementation of the Riemannian potato artifact detector provided by the authors in the covariance toolbox for MATLAB (github.com/alexandrebarachant/covariancetoolbox). Visual comparison of scoring obtained by RPs and by the benchmark is provided in Figure 5 and Figure 6. Detection results were compared with scoring provided by an expert, and the three statistical metrics presented in the previous section were computed as well as percentage of data labeled as artifacts by methods. Each recording of the Dream dataset contains a mix of sleep stages and awake EEG. Some of the presented sleep stages have a short duration and do not include artifacts. Testing results grouped for such EEG types would not be representative. Therefore, metric values for the Dreams dataset were obtained for the entire recordings. Recordings in the InSleep dataset contain activity of a single stage. That allowed us to access the method performance on separate sleep stages and wakefulness. Table 2 reports the obtained results for the benchmark, and Table 3 contains values for the proposed method. Cohen’s kappa for both methods on all datasets is shown in Figure 7.

The proposed method, RPs, performed better than the benchmark in terms of FDR for the Dreams dataset (*p*-value <0.05) and InSleep REM and non-REM 2 data (*p*-value <0.05 and *p*-value <0.01, respectively). With respect to sensitivity, RPs did not outperform the benchmark. Finally, RPs significantly outperformed the benchmark method on non-REM 2 (*p*-value <0.01) and non-REM 3 (*p*-value < 0.05) in terms of agreement with the expert scoring. A similar trend is observed for the InSleep dataset, as shown in Figure 7 (*p*-value <0.1).

**Figure 8:**
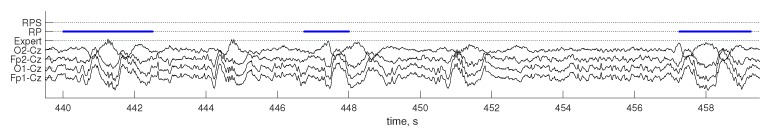
Dreams dataset. Lines represent the expert scoring (Expert), results of the benchmark (RP), and the proposed (RPS) methods.

Visual inspection in the Dreams dataset revealed that the benchmark method tended to detect incorrectly as artifacts characteristic EEG patterns of non-dominant sleep stages such as high amplitude delta-waves corresponding to non-REM 3 activity in recordings with a dominant non-REM 2 sleep stage, as illustrated in Figure 8.

**Figure 9:**
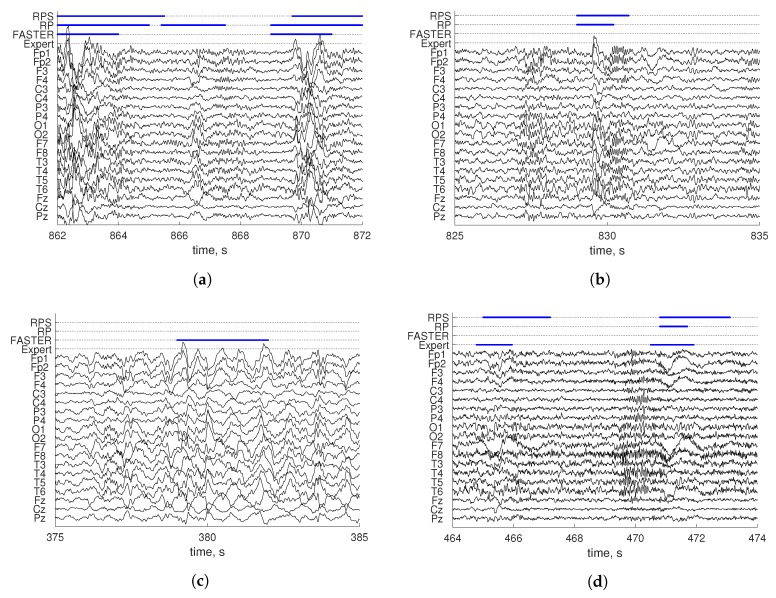
Expert scoring (Expert), results of the FASTER (FASTER), benchmark (RP), and the proposed (RPS) methods of different EEG patterns in delta waves (**a**), k-complex in non-REM 2 (**b**), delta waves in non-REM 3 (**c**), and ocular artifact in REM (**d**).

FDR values obtained on InSleep non-REM 2 indicated a big proportion of false positive events. Both methods incorrectly detected injections of non-REM 3 sleep in non-REM 2. This activity is defined as high amplitude slow waves lasting 1–5 s. An example is provided in Figure 9a. Such events account for a small proportion of the recording for which the methods do not appear adapted. Furthermore, the non-REM 2 sleep data also contained K-complexes and sleep spindles characterized as a single high-amplitude delta wave and sigma bursts, respectively. An example is depicted in Figure 9b. A wide range of patterns of such events complicates the detection for both methods. Moreover, artifacts in stages non-REM 2 and non-REM 3 occurred rarely according to Table 1; this made the metrics values for these datasets more sensitive to false detections.

As we have mentioned, RPs utilizes 1-s epochs, which is a standard window length for Riemannian geometry analysis. We have also applied RPs using 2-s and 4-s epoch lengths to all datasets to test whether longer epoch lengths are more suitable for capturing the dynamics of slow waves. The obtained results are presented in Table 4 and Table 5 respectively. The results showed no significant increase in *K* in all datasets. In fact, a significant decrease in *K* was observed for wakefulness and REM InSleep (*p*-value < 0.01). Indeed, the number of events corresponding to normal slow wave activity and incorrectly determined as artifacts in non-REM 2 and 3 decreased. However, using such modifications decreased the number of detected events in general and increased the number of false results. This is caused by an increased number of missed small artifacts. Moreover, the detected events were too long, which led to an increase of the FP value and, consequently, FDR.

Additionally, we have applied FASTER [25] to all datasets. This method rejects epochs of a single-channel EEG based on calculated thresholds. The process is repeated for each channel. Mean, variance, amplitude range, and mean gradient are used for decision making. The results are presented in Table 6. The proposed RPs method significantly outperformed FASTER in *K* for all datasets (*p*-value <0.05 for InSleep non-REM 3, *p*-value <0.01 for others). Moreover, RPs achieves significantly better Se in InSleep wakefulness (*p*-value <0.01), REM, and non-REM 2 (*p*-value <0.05 for both datasets) and significantly smaller FDR for Dreams, InSleep non-REM 2 and 3 (*p*-value <0.01, *p*-value <0.05, and *p*-value <0.01, respectively). The method performed well in the detection of movement artifacts. However, often, it incorrectly detected a high amplitude delta in non-REM 2 (Figure 9a) and non-REM 3 (Figure 9c) as an artifact. Furthermore, it missed many short and small-amplitude artifacts (Figure 9d).

For the estimation of time consumption, we ran FASTER and RPs on 15-min 19-channel recordings. A Windows 10 Pro computer with an Intel Core i7 2.40-GHz CPU and 16 GB of RAM was used. Testing of FASTER, excluding the ICA decomposition, which was very time consuming, was performed using MATLAB R2014b. The processing time was 5.44±0.01 s per recording. Testing of RPs was performed using Python 2.7. The processing time of RPs was 43.86–112.63 s per recording depending on the number of constructed clusters. Cluster construction took 0.03 s for a single cluster and 68.72 s for the construction of six clusters. Testing on the Dreams dataset resulted in 1–4 clusters. The number of clusters in the InSleep dataset varied in the range of 1–6 for awake and REM EEG and in the range of 1–4 for non-REM 2 and 3 activity. The number of clusters did not depend on the number of channels, but only on the presence of different activities, which could be distinguished in terms of the Riemannian geometry of the data.

## 5. Discussion

The performance of RPs was compared to a previously-developed artifact detection method based on Riemannian geometry. Testing on rest-state awake data confirmed the effectiveness of both RPs and the benchmark approaches. The results obtained for sleep data proved that the proposed method was more favorable. The reason why RPs outperformed the benchmark in sleep data is that the multiple cluster construction was adapted to the variability of normal spatial patterns in sleep stages. This resulted in a reduction of false detections and improved agreement with the expert. The method was fully unsupervised and adaptive; therefore, detection was dependent only on the input data and could be completely automated. Testing results proved that on the data we considered, a 1-s epoch length was an optimal choice for RPs. Furthermore, the proposed method is scalable and may be applied for any number of channels. Nevertheless, using highly-correlated signals makes the application of the method cumbersome because the estimated covariance matrices may be badly conditioned. Therefore, in the case of a very large number of EEG channels, a dimensionality-reduction pre-processing step is recommended. To do this, methods inspired by the Riemannian geometry of SPD matrices may be used; see [32,33].

In comparison to FASTER, which relies on statistical properties in separate channels, the methods based on Riemannian geometry achieved better performance on all datasets. FASTER missed too many artifacts, mostly small ocular and short electrode artifacts. Nevertheless, all tested methods equally performed well on the detection of high frequency/amplitude artifacts. Such artifacts prevailed in wakefulness, which allowed FASTER to achieve better performance among all datasets therein. For sleep, FASTER overestimated artifact activity, which lad to low performance. In non-REM 2, it detected only injections of non-REM 3 activity, which noticeably affected the metric values. All methods detected injections of non-REM 3 sleep into non-REM 2 as an artifact. Even if such events were not labeled by the expert as artifacts, their elimination led to improvement of the spectral characteristics of non-REM 2 activity. As for computational efforts, FASTER significantly outperformed RPs, since it utilized simple signal statistics, whereas RPs was based on eigenvalue estimations for computing Riemannian distances. The time required by the RPs method, however, is low in absolute terms and very acceptable for any practical purpose.

This study shows once more that automatic artifact detection in sleep EEG is a challenging task and demonstrates the low efficiency of methods developed for awake EEG. Many studies addressing this problem have proposed methods for artifact detection without information about sleep stages [22]. However, EEG investigation pipelines in modern sleep research laboratories include analysis of sleep data with already labeled sleep stages. Some recent studies have proposed artifact detection based on analysis of 20- and 30-s epochs [23]. Evaluation using 20-s (30-s) epochs is required for data investigation at a macro level in sleep scoring by humans, whereas for an automatic artifact rejection, it can be performed at a micro-level, and it is preferable to consider smaller epochs. A way to make the automatic method more comparable to human scoring would be to reject whole 20-s (30-s) segments if the majority of 1-s epochs forming them were rejected. Moreover, sleep disorders increase the inter-subject variability of the EEG, as well as the number of artifacts, which makes the task of artifact detection even more demanding. For these and other situations, a fully-unsupervised and adaptive strategy such as RPs is theoretically appealing.

### Limitations and Future Work

The RPs method we have proposed has several disadvantages and therefore can be further improved. First, infrequent EEG patterns might not be represented in the clusters of artifact-free activity due to the cluster construction procedure. Thus, they might be incorrectly detected as artifacts. An improvement could be achieved by setting an automatic method for identification of expected patterns like K-complexes and post-processing analysis. Moreover, the method seems to fail in rejecting artifacts that are not widely distributed on the scalp, such as muscular or cardiac artifacts. This may be obviated by constructing smaller potatoes that include only a small number of channels and by combining the rejection of all the potatoes (Riemannian potato fields), which is currently under investigation. The method was not tested on high-density EEG. In this case, the problem of highly-correlated signals in high-density EEG must be carefully considered. Future work will also be focused on improvements of the method in order to eliminate false detections of K-complexes and sleep spindles. The application of the method is currently under consideration for semi-automatic artifact rejection at the National Institute of Mental Health of Czech Republic. At this stage, probability scoring will be provided along with data to support manual investigation. Additionally, the method will be extended to multimodal PSG recordings. Other directions of future research include application of the proposed method and analysis of the obtained clusters for automatic sleep stage identification using supervised machine learning approaches.

## 6. Conclusions

The study presents an unsupervised multichannel artifact detection method for sleep EEG based on Riemannian geometry. The presented method forms clusters of artifact-free EEG data considering their spatial patterns. Application of the proposed method to sleep recordings brings significant benefits. The method is scalable, fully unsupervised and adaptive, independent of artifact types, and the outcome is easily interpreted. In comparison to the Riemannian potato artifact detector, it demonstrates better performance on complex sleep data in terms of agreement with human scoring and reduces the number of events incorrectly identified as artifacts. The RPs toolbox is available at https://gitlab.ciirc.cvut.cz/open-source/rps.

## Figures and Tables

**Table 1:** Dataset details.

Dataset	Sleep Stage	Number of Subjects	Length (minutes)	Artifact Rate (%)
Dreams		12	15	9.38±9.84
InSleep	Wakefulness	14	15.38±2.26	18±9.9
	REM	12	13.5±1.88	7.43±5.82
	Non-REM 2	10	15.3±1.77	4.07±5.33
	Non-REM 3	8	14.75±2.76	1.97±1.43

**Table 2:** Performance of the benchmark artifact detection method.

Dataset	Sleep Stage	*K*	Se	FDR	Artifact Rate (%)
Dreams		0.58±0.2	0.79±0.19	0.4±0.21	12.23±13.71
InSleep	Wakefulness	0.84±0.09	0.91±0.08	0.16±0.07	15.2±8.24
	REM	0.75±0.24	0.88±0.12	0.28±0.24	8.3±7.14
	Non-REM 2	0.40±0.24	0.77±0.21	0.62±0.41	9.12±13.53
	Non-REM 3	0.54±0.17	0.62±0.29	0.37±0.21	1.58±1.52

**Table 3:** Performance of RPs artifact detection.

Dataset	Sleep Stage	*K*	Se	FDR	Artifact Rate (%)
Dreams		0.74±0.17	0.78±0.18	0.24±0.22	7.49±7.65
InSleep	Wakefulness	0.85±0.09	0.9±0.08	0.12±0.08	16.6±7.62
	REM	0.82±0.11	0.86±0.12	0.18±0.13	9.47±8.22
	Non-REM 2	0.50±0.37	0.84±0.17	0.55±0.38	9.06±5.81
	Non-REM 3	0.68±0.22	0.68±0.26	0.22±0.22	1.42±1.5

**Table 4:** Performance of RPs artifact detection with a 2-s epoch length.

Dataset	Sleep Stage	*K*	Se	FDR	Artifact Rate (%)
Dreams		0.71±0.17	0.85±0.1	0.31±0.17	12.17±13.29
InSleep	Wakefulness	0.77±0.13	0.85±0.1	0.2±0.14	18.75±8.17
	REM	0.72±0.14	0.8±0.19	0.25±0.19	8.99±5.31
	Non-REM 2	0.53±0.41	0.79±0.21	0.49±0.44	9.75±7.78
	Non-REM 3	0.67±0.26	0.67±0.26	0.26±0.27	2.74±2.08

**Table 5:** Performance of RPs artifact detection with a 4-s epoch length.

Dataset	Sleep Stage	*K*	Se	FDR	Artifact Rate (%)
Dreams		0.52±0.16	0.8±0.14	0.54±0.16	15.12±15.27
InSleep	Wakefulness	0.68±0.11	0.84±0.1	0.32±0.14	20.75±8.62
	REM	0.53±0.16	0.66±0.2	0.46±0.2	9.59±7.92
	Non-REM 2	0.46±0.38	0.77±0.19	0.57±0.38	9.52±7.74
	Non-REM 3	0.55±0.23	0.62±0.3	0.46±0.24	2.87±2.18

**Table 6:** Performance of the FASTER artifact detection method.

Dataset	Sleep Stage	*K*	Se	FDR	Artifact Rate (%)
Dreams		0.31±0.22	0.81±0.28	0.66±0.26	24.9±11.55
InSleep	Wakefulness	0.58±0.21	0.52±0.24	0.09±0.14	6.45±6.14
	REM	0.46±0.21	0.57±0.24	0.45±0.27	8.64±6.3
	Non-REM 2	0.28±0.25	0.43±0.36	0.72±0.25	3.46±2.12
	Non-REM 3	0.41±0.21	0.66±0.34	0.64±0.16	3.29±1.57

## Abbreviations

The following abbreviations are used in this manuscript:

BCI	Brain-computer interface
BSS	Blind source separation
EEG	Electroencephalogram
FASTER	Fully-automated statistical thresholding for EEG artifact rejection
FN	False negative
FP	False positive
ICA	Independent component analysis
non-REM	Non-rapid eye movement (sleep stage)
PSG	Polysomnography
REM	Rapid eye movement (sleep stage)
RPs	Riemannian potatoes
SCM	Sample covariance (matrix)
SPD	Symmetric positive-definite (matrix)
TN	True negative
TP	True positive
